# Large Asymptomatic Schwannoma of the Tongue: An Unusual Case Report With Clinical, Radiologic, and Histopathological Correlation

**DOI:** 10.7759/cureus.99076

**Published:** 2025-12-12

**Authors:** Deepinder Pal Singh Sodhi, Palveen Kaur, Gursimrat Brar, Sumit Prinja, Gurshinder Pal Singh

**Affiliations:** 1 Department of Otorhinolaryngology, Guru Gobind Singh Medical College and Hospital, Faridkot, IND; 2 Department of Oral and Maxillofacial Surgery, Dasmesh Institute of Research and Dental Sciencess, Faridkot, IND

**Keywords:** benign, excision, neoplasm, neurilemmoma, schwannoma, tongue

## Abstract

Schwannomas of the oral cavity are rare benign tumors, with the tongue being the most frequently affected site. This case describes a 22-year-old male presenting with a large, well-circumscribed mass on the dorsum of the tongue that remained entirely asymptomatic despite its considerable size. Clinical examination and non-contrast computed tomography revealed a localized hypodense lesion without infiltration. Complete transoral excision was performed, and histopathological analysis demonstrated classic Antoni A and B patterns with Verocay bodies, confirming the diagnosis of schwannoma. Postoperative healing was uneventful, with no functional deficits or recurrence during follow-up. This case highlights the importance of considering schwannoma in the differential diagnosis of tongue masses, as the clinical features may resemble fibroma, neurofibroma, or other benign soft-tissue tumors. Early identification and complete surgical removal ensure an excellent prognosis and preservation of tongue function.

## Introduction

Schwannomas, also known as neurilemmomas, are benign, slow-growing, encapsulated tumors arising from the Schwann cells of the peripheral nerve sheath. They may originate from any myelinated nerve, including the cranial, spinal, and autonomic nerves, except the optic and olfactory nerves, which lack Schwann cells [1]. Approximately 25-45% of all schwannomas occur in the head and neck region, making them relatively common locations for these neoplasms [2]. However, intraoral schwannomas are distinctly uncommon, representing only 1%-12% of all head and neck schwannomas [3]. Among intraoral sites, the tongue is reported to be the most frequently involved, followed by the floor of the mouth, palate, buccal mucosa, gingiva, lips, and vestibule [1,4]. Early reviews, such as the landmark analysis by Gallo et al., identified the tongue as the predominant site among the studied oral schwannoma cases [5]. The clinical presentation depends largely on lesion size and location; smaller anterior tongue tumors may remain asymptomatic, whereas posterior or larger masses may interfere with speech, mastication, or airway patency.

Histopathological examination remains the diagnostic gold standard, characterized by the presence of Antoni A and B areas and Anti-S100 protein [3,6]. Complete surgical excision is curative in most cases, and recurrence is exceedingly rare [7]. The present case report describes a rare presentation of a large schwannoma located on the dorsum of the tongue, detailing its clinical characteristics, imaging features, surgical management, and histopathological confirmation, thereby contributing to the limited literature on lingual schwannomas and emphasizing the importance of early diagnosis and appropriate intervention.

## Case presentation

A 22-year-old male was admitted to the Department of Otorhinolaryngology, Guru Gobind Singh Medical College and Hospital, Faridkot, Punjab, with concerns regarding a slowly enlarging mass on the dorsum of his tongue. The patient first noticed the swelling several months earlier; however, sought medical attention only when he became aware of the gradual increase in size. There was no history of pain, dysphagia, dysarthria, bleeding, altered taste sensation, or airway difficulty. The patient denied any preceding trauma or infection in the region. His medical, dental, and family histories were non-contributory, and no habits, such as tobacco use, were reported.

**Figure 1:**
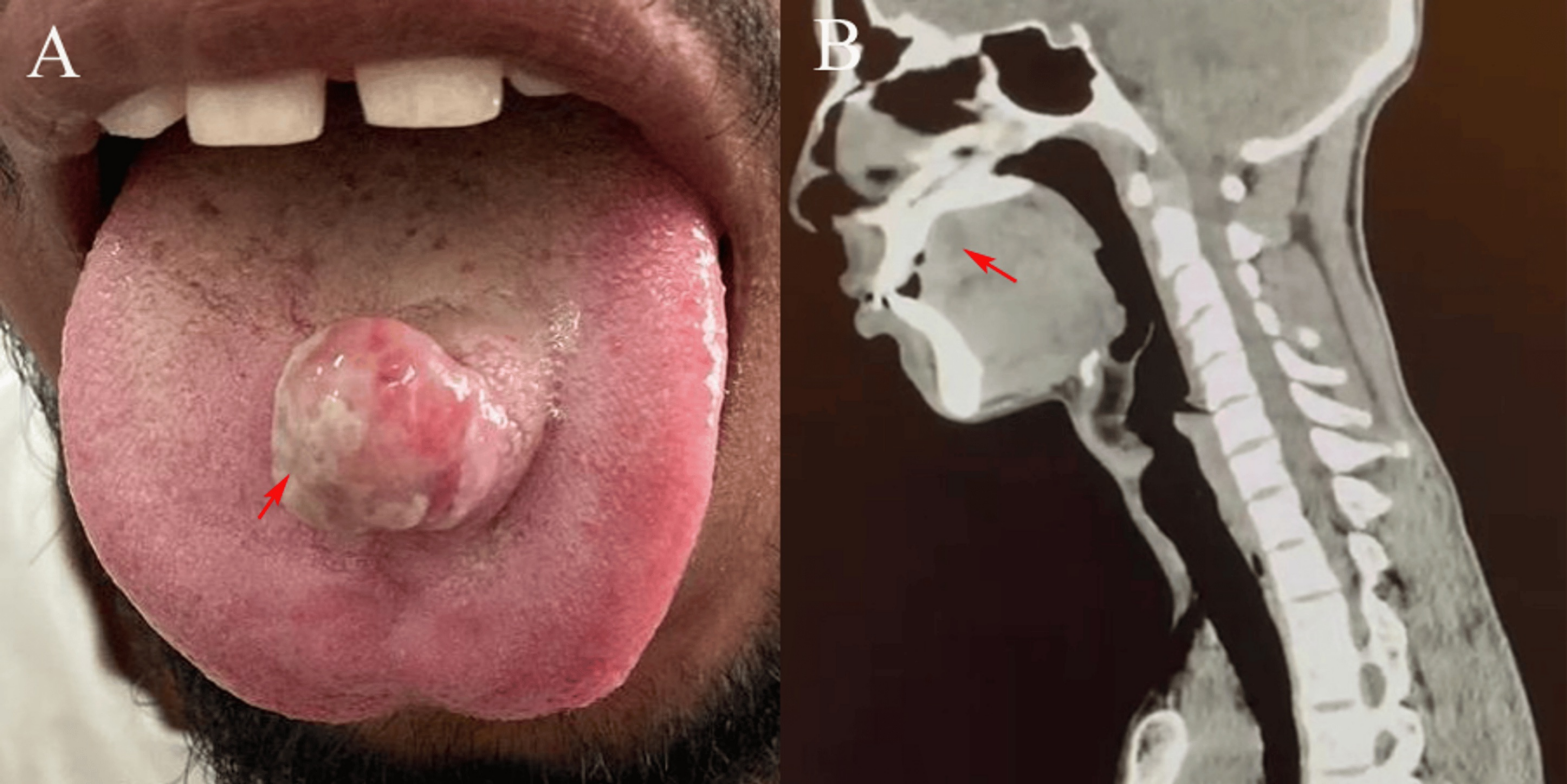
Clinical and saggital CT images of the patient. (A) Clinical photograph showing a lobulated, firm, well-circumscribed 4–5 cm mass on the mid-dorsal surface of the anterior two-thirds of the tongue (arrow). (B) Sagittal non-contrast computed tomography of the neck demonstrating a well-defined hypodense lesion on the tongue dorsum (arrow) with no invasion into adjacent musculature or deeper structures.

An extraoral examination revealed no cervical lymphadenopathy or facial asymmetry. Intraoral examination revealed a lobulated, firm, well-circumscribed mass measuring approximately 4-5 cm on the mid-dorsal surface of the anterior two-thirds of the tongue. The mucosa overlying the lesion was intact and normal in color, with no ulceration. The mass was nontender and exhibited limited mobility relative to the surrounding tissue. Tongue movements, including protrusion and lateralization, were preserved, and taste sensation appeared normal. No evidence of neurological deficits was detected, making it difficult to clinically identify the origin of the nerve (Figure 1A). Routine hematological investigations were within normal limits. A non-contrast computed tomography (NCCT) scan of the neck revealed a well-defined hypodense lesion on the dorsum of the tongue with no invasion into the adjacent musculature or deeper structures (Figure 1B). The lesion appeared encapsulated and surgically accessible, without evidence of regional spread. Based on clinical and radiological findings, the patient was scheduled for surgical excision under general anesthesia. The transoral approach was used in this study. The lesion was exposed, and complete excision (in toto) was achieved with preservation of the surrounding musculature and neurovascular structures. The surgical field was hemostatic and the postoperative course was uneventful. The patient was discharged after receiving routine postoperative instructions.

**Figure 2:**
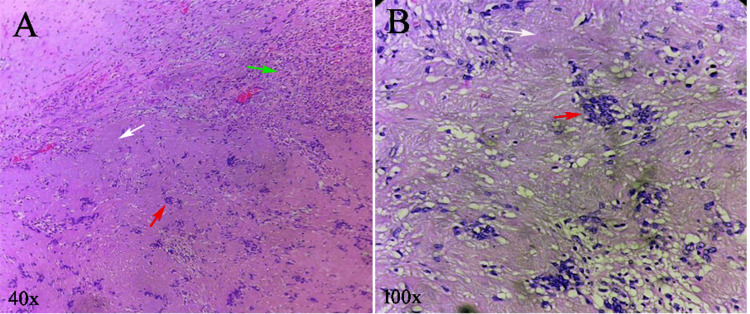
Hematoxylin and eosin (H&E)-stained sections of schwannoma. (A) Low-power view (40x) shows classic biphasic pattern showing compact hypercellular Antoni A areas (white arrow), loosely arranged hypocellular Antoni B areas (green arrow), and focal Verocay body formation (red arrow). (B) High-power view (100x) shows typical Antoni A region demonstrating palisading spindle-shaped Schwann cells arranged around acellular eosinophilic Verocay bodies (red arrow) with surrounding myxoid Antoni B tissue (white arrow).

Grossly, the excised specimen was firm and encapsulated. Histopathological examination revealed a classic biphasic pattern consisting of hypercellular Antoni A areas with palisading spindle cells and prominent Verocay bodies, along with hypocellular Antoni B areas arranged in a myxoid stroma. These features confirmed the diagnosis of a schwannoma (Figure 2).

**Figure 3:**
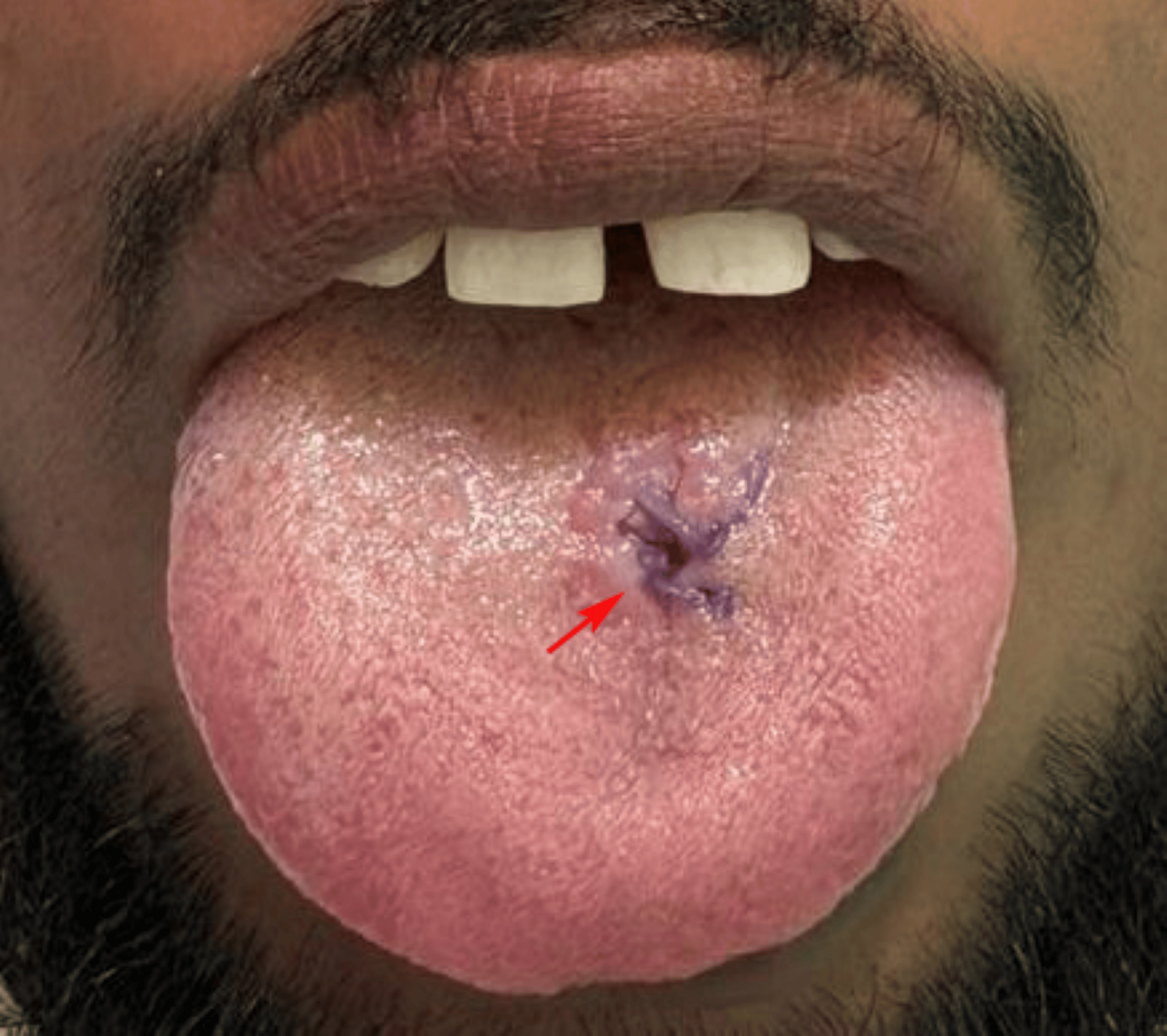
Healed area on dorsum surface of tongue.

Malignant features were not observed. The patient was reviewed regularly, and the two-week postoperative follow-up showed excellent healing, with no residual swelling, functional limitation, or sensory disturbance. At the subsequent follow-up visits, the patient remained asymptomatic, and no signs of recurrence were observed (Figure 3).

## Discussion

Schwannomas of the oral cavity are rare benign neoplasms originating from Schwann cells of the peripheral nerve sheaths. Although 25-45% of schwannomas arise in the head and neck region, their intraoral occurrence remains uncommon, accounting for only 1-12% of all cases [1-4]. Among intraoral sites, the tongue is the most frequently affected location, as established in classical reports by Gallo et al. [5] and later supported by Lira et al. [8], who reaffirmed that the tongue is the predominant site because of its rich sensory and motor innervation. The present case further adds to the body of evidence highlighting the tongue, especially its anterior dorsum, as a favorable site for schwannoma development.

Clinically, lingual schwannomas are usually slow-growing, painless, firm nodules with well-defined margins, which is consistent with our patient’s presentation. Bhola et al. [9] described similar features in a pediatric lingual schwannoma, emphasizing the asymptomatic nature of these tumors until they reach a size that interferes with speech and mastication. Lesion size plays a significant role in symptomatology: masses smaller than 2.5 cm often remain asymptomatic, whereas posteriorly located tumors or those exceeding 3 cm may cause obstructive or functional symptoms. Interestingly, despite the relatively large size of the lesion (4-5 cm), the patient did not exhibit dysphagia, airway compromise, or articulation difficulties, supporting previous observations that symptom severity does not always correlate directly with tumor size [7].

Radiographically, magnetic resonance imaging (MRI) is generally preferred for soft tissue characterization of tongue lesions [10]; however, in this case, an NCCT scan adequately demonstrated a well-circumscribed hypodense mass without infiltrative features, consistent with the benign schwannoma described by Alrohaimi et al. [11]. Histopathologically, schwannomas are encapsulated tumors displaying characteristic biphasic Antoni A and Antoni B patterns with Verocay bodies, all of which were identified in this case [6]. This case involved a 22-year-old male patient, similar to those described in reports by Lira et al. [8] and Hsu et al. [12].

Complete surgical excision is the treatment of choice. Transoral removal is widely accepted for accessible anterior tongue lesions, as supported by previous reports [3,7,13]. Recurrence is extremely rare following complete resection, given the typically encapsulated nature of these tumors. Our patient demonstrated excellent postoperative healing, with no functional impairment or recurrence on follow-up, consistent with the outcomes observed in previous reports [3,8].

A major limitation of this case is the absence of MRI imaging, which could have provided superior soft-tissue contrast and better delineation of the lesion’s relationship to the intrinsic tongue musculature. Additionally, long-term follow-up is still ongoing. While recurrence is rare, extended monitoring would offer a more comprehensive assessment of treatment success. An acid S-100 protein test was not performed, as the hematoxylin-eosin-stained sections conclusively confirmed the diagnosis. Finally, the nerve of origin could not be identified, which is a common but notable limitation in lingual schwannoma cases owing to overlapping innervation.

This case emphasizes the need for clinicians, especially dentists, oral surgeons, and otorhinolaryngologists, to consider schwannomas in the differential diagnosis of well-circumscribed, asymptomatic tongue masses. Early recognition facilitates timely imaging, appropriate surgical planning, and the prevention of potential airway or functional complications. This case also highlights the effectiveness of transoral excision for accessible tongue lesions, reinforcing its status as a minimally invasive, function-preserving approach.

## Conclusions

Schwannoma of the tongue is an uncommon benign neoplasm, and although it is more frequently documented in young adults, its overall rarity means that it is often overlooked in routine clinical evaluation. This case adds to the limited literature by presenting a large asymptomatic schwannoma on the dorsum of the tongue, an entity that may clinically mimic fibroma, neurofibroma, salivary gland tumors, leiomyoma, rhabdomyoma, lymphangioma, or hemangioma. As demonstrated here, a definitive diagnosis relies on characteristic histopathological features. Complete surgical excision remains curative, with no recurrence observed during the follow-up. Malignant transformation is exceedingly rare, reinforcing the excellent prognosis associated with its timely diagnosis and management.
